# Supplementary material for: Mitochondrial surface coating with artificial lipid membrane improves the transfer efficacy
Source: Commun Biol. 2022 Jul 25;5:745. doi: 10.1038/s42003-022-03719-9 (PMC9314363; doi:10.1038/s42003-022-03719-9)
Supplement: Supplementary file 2 — Supplementary information [file 42003_2022_3719_MOESM2_ESM.pdf]

Figure 2d

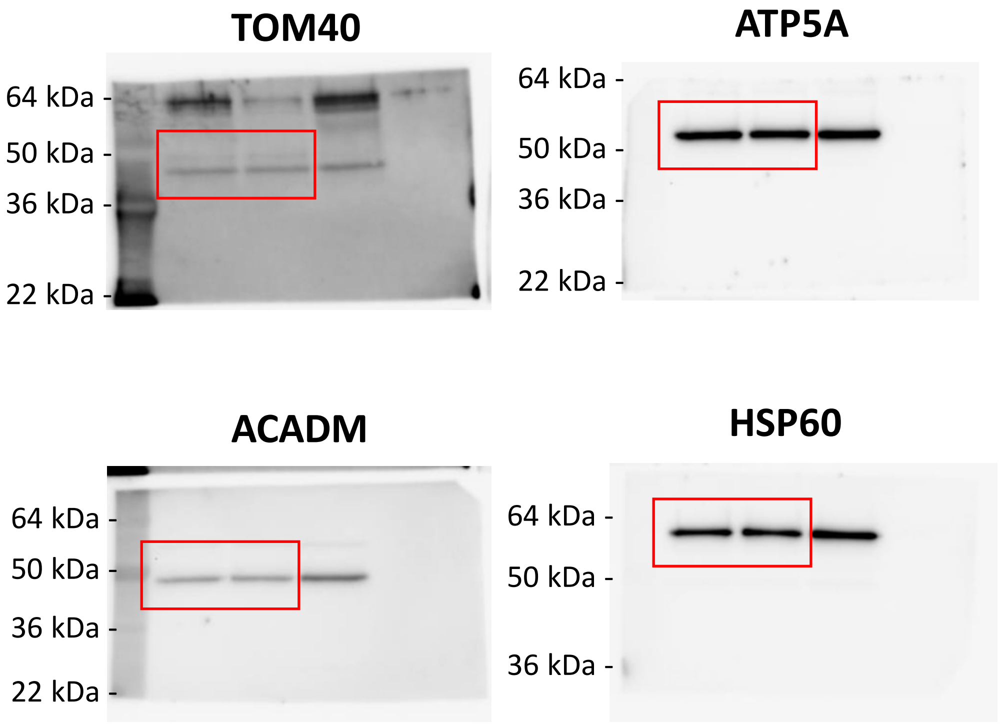

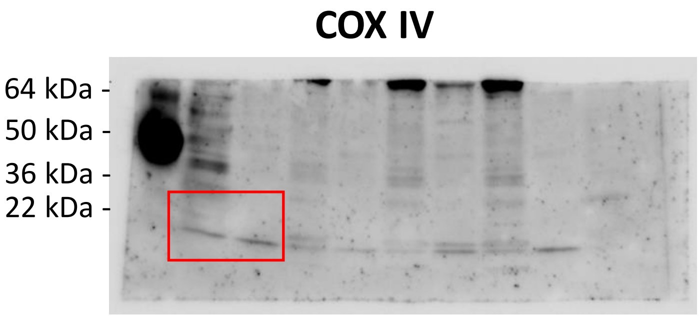

Figure 3c

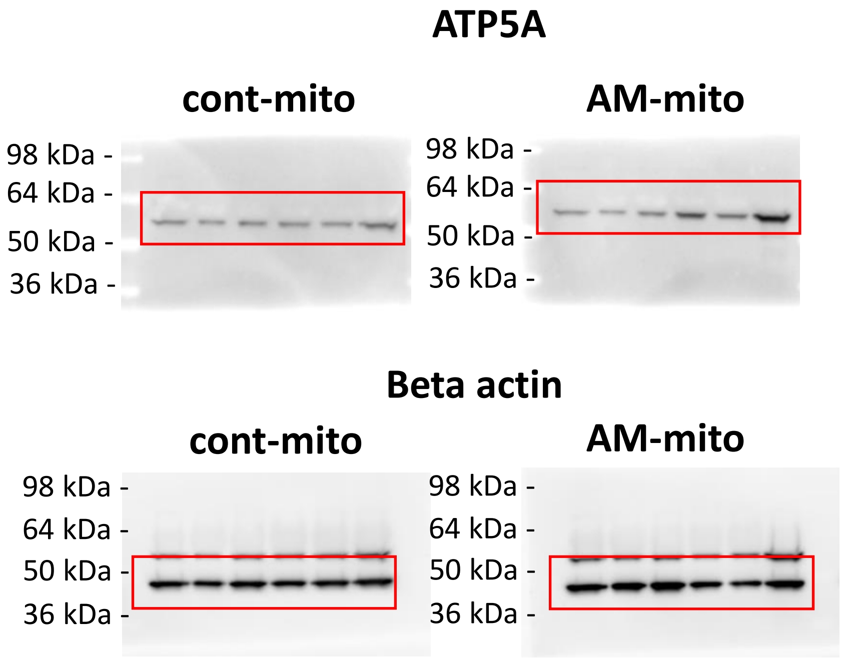

Supplementary Figure 1: Uncropped western blot images. Red rectangles in TOM40, ATP5A, ACADM, HSP60, COXIV or ATP5A, beta-actin were shown in Figure 2d or 3c, respectively.
