# Supplementary material for: Mitochondrial surface coating with artificial lipid membrane improves the transfer efficacy
Source: Commun Biol. 2022 Jul 25;5:745. doi: 10.1038/s42003-022-03719-9 (PMC9314363; doi:10.1038/s42003-022-03719-9)
Supplement: Supplementary file 3 — Description of additional supplementary files [file 42003_2022_3719_MOESM3_ESM.pdf]

Description of Additional Supplementary Files

File name: Supplementary Data 1

Description: The source data for all the graphs presented in figures
