# Supplementary material for: Mitochondrial surface coating with artificial lipid membrane improves the transfer efficacy
Source: Commun Biol. 2022 Jul 25;5:745. doi: 10.1038/s42003-022-03719-9 (PMC9314363; doi:10.1038/s42003-022-03719-9)
Supplement: Supplementary file 5 — Reporting Summary [file 42003_2022_3719_MOESM5_ESM.pdf]

nature portfolio

Corresponding author(s): Kazuhide Hayakawa

Last updated by author(s): Jun 21, 2022

Reporting Summary

Nature Portfolio wishes to improve the reproducibility of the work that we publish. This form provides structure for consistency and transparency in reporting. For further information on Nature Portfolio policies, see our Editorial Policies and the Editorial Policy Checklist.

Statistics

For all statistical analyses, confirm that the following items are present in the figure legend, table legend, main text, or Methods section.

n/a	Confirmed	
☐	☒	The exact sample size (*n*) for each experimental group/condition, given as a discrete number and unit of measurement
☐	☒	A statement on whether measurements were taken from distinct samples or whether the same sample was measured repeatedly
☐	☒	The statistical test(s) used AND whether they are one- or two-sided<br>*Only common tests should be described solely by name; describe more complex techniques in the Methods section.*
☐	☒	A description of all covariates tested
☐	☒	A description of any assumptions or corrections, such as tests of normality and adjustment for multiple comparisons
☐	☒	A full description of the statistical parameters including central tendency (e.g. means) or other basic estimates (e.g. regression coefficient) AND variation (e.g. standard deviation) or associated estimates of uncertainty (e.g. confidence intervals)
☐	☒	For null hypothesis testing, the test statistic (e.g. *F*, *t*, *r*) with confidence intervals, effect sizes, degrees of freedom and *P* value noted<br>*Give P values as exact values whenever suitable.*
☒	☐	For Bayesian analysis, information on the choice of priors and Markov chain Monte Carlo settings
☒	☐	For hierarchical and complex designs, identification of the appropriate level for tests and full reporting of outcomes
☐	☒	Estimates of effect sizes (e.g. Cohen's *d*, Pearson's *r*), indicating how they were calculated

Our web collection on statistics for biologists contains articles on many of the points above.

Software and code

Policy information about availability of computer code

Data collection	No softwares were used to collect data.
Data analysis	Mitochondrial quality after coating was assessed by particle size and zeta potential analysis by Delsa Nano, western blot and FACS.

For manuscripts utilizing custom algorithms or software that are central to the research but not yet described in published literature, software must be made available to editors and reviewers. We strongly encourage code deposition in a community repository (e.g. GitHub). See the Nature Portfolio guidelines for submitting code & software for further information.

Data

Policy information about availability of data

All manuscripts must include a data availability statement. This statement should provide the following information, where applicable:

- Accession codes, unique identifiers, or web links for publicly available datasets
- A description of any restrictions on data availability
- For clinical datasets or third party data, please ensure that the statement adheres to our policy

The authors declare that the data supporting the findings of this study are available within the paper and its supplementary information files or from the corresponding author upon reasonable request.

Field-specific reporting

Please select the one below that is the best fit for your research. If you are not sure, read the appropriate sections before making your selection.

☒ Life sciences ☐ Behavioural & social sciences ☐ Ecological, evolutionary & environmental sciences

For a reference copy of the document with all sections, see nature.com/documents/nr-reporting-summary-flat.pdf

Life sciences study design

All studies must disclose on these points even when the disclosure is negative.

Sample size	Sample size was predetermined using the software available online: https://www.danielsoper.com/statcalc/calculator.aspx?id=47 The calculation was based on Cohen's d value where SD and average were estimated from our historical and preliminary data.
Data exclusions	All data were included.
Replication	For in vitro experiments, each experiment was repeated at least 3 times.
Randomization	All experiments followed standard protocols for randomization of group assignment via 4 number lottery draw, allocation concealment, blinding of operators, blinding of measurements, blinding of analyses.
Blinding	All procedures and measurements were performed in a blinded and randomized fashion.

Reporting for specific materials, systems and methods

We require information from authors about some types of materials, experimental systems and methods used in many studies. Here, indicate whether each material, system or method listed is relevant to your study. If you are not sure if a list item applies to your research, read the appropriate section before selecting a response.

Materials & experimental systems

n/a	Involved in the study	
☐	☒	Antibodies
☒	☐	Eukaryotic cell lines
☒	☐	Palaeontology and archaeology
☐	☒	Animals and other organisms
☒	☐	Human research participants
☒	☐	Clinical data
☒	☐	Dual use research of concern

Methods

n/a	Involved in the study	
☒	☐	ChIP-seq
☐	☒	Flow cytometry
☒	☐	MRI-based neuroimaging

Antibodies

Antibodies used	anti-β-actin (1:1,000, Sigma-aldrich A5441), anti-TOM40 (1:200, Santacruz, sc-11414), anti-ATP5A (1:500, Abcam, ab14748), anti-ACADM (1:500, Abcam, ab92461), and anti-HSP60 (1:500, Abcam, ab46798).
Validation	Antibodies used in this study were validated by comparing with the no-primary and no-secondary controls.

Animals and other organisms

Policy information about studies involving animals; ARRIVE guidelines recommended for reporting animal research

Laboratory animals	E18 embryo of Sprague-Dawley rats or E18 emtryo of C57BL/6J mice were used in this study.
Wild animals	No wild animals were used in this study.
Field-collected samples	No field-collected were used in this study.
Ethics oversight	All experiments were performed following an institutionally approved protocol in accordance with National Institutes of Health guidelines and with the United States Public Health Service's Policy on Human Care and Use of Laboratory Animals and following Animals in Research: Reporting In vivo Experiments (ARRIVE) guidelines.

Note that full information on the approval of the study protocol must also be provided in the manuscript.

Flow Cytometry

Plots

Confirm that:

☒ The axis labels state the marker and fluorochrome used (e.g. CD4-FITC).

☒ The axis scales are clearly visible. Include numbers along axes only for bottom left plot of group (a 'group' is an analysis of identical markers).

☒ All plots are contour plots with outliers or pseudocolor plots.

☒ A numerical value for number of cells or percentage (with statistics) is provided.

Methodology

Sample preparation	To coat microbeads (F13838, 1 µm, ThermoFisherScientific), liposomes compose of 7:3 molar ratio of L-alpha-phosphatidylcholine : L-±-phosphatidylserine (Encapsula NanoScience), or isolated mitochondria, the inverted emulsion method was used. Mitochondria were isolated from intact cerebral cortex of male C57BL/6J mice (12-14 weeks) and we prepared mitochondrial suspension in mitochondria buffer consist of 10 mM HEPES pH 7.5, 250 mM Sucrose, 1 mM ATP, 0.1 mM ADP, 5 mM Sodium succinate, 2 mM Dipotassium phosphate, and 1% polyvinyl alcohol. Mitochondrial suspension (20 µg/5 µL) was mixed with 100 µL of mineral oil containing 1 mM DOTAP/DOPE (1:1), followed by generating water/oil (W/O) emulsion by gently pipetting 10 times. The W/O emulsion was transferred onto 150 µL of mineral oil containing 1mM DOTAP/DOPE (1:1) on 500 µL of PBS prepared in 1.5 mL tube at least 30 minutes before adding W/O emulsion. Five minutes after slowly adding W/O emulsion, the test tubes were spinned by the centrifugation at 4,000g for 10 minutes at 4oC. Supernatant was discarded and the pellet was carefully resuspended in mitochondria buffer and washed one time with a centrifugation with 4,000g for 5 minutes at 4oC. Mitochondrial quality after coating was assessed by particle size and zeta potential analysis by Delsa Nano, western blot and FACS.
Instrument	BD Fortessa
Software	FlowJo and Flowing Software 2 (https://bioscience.fi/services/cell-imaging/flowing-software/)
Cell population abundance	Cell isolation was not performed.
Gating strategy	FACS analysis was performed to determine Mitotracker DR or DJ1 monomer and dimer using BD Fortessa with a no labeled control for determining appropriate gates, voltages, and compensations required in multivariate flow cytometry.

☒ Tick this box to confirm that a figure exemplifying the gating strategy is provided in the Supplementary Information.
